# Does peer feedback for teaching GPs improve student evaluation of general practice attachments? A pre-post analysis

**DOI:** 10.3205/zma001518

**Published:** 2021-11-15

**Authors:** Michael Pentzek, Stefan Wilm, Elisabeth Gummersbach

**Affiliations:** 1 Heinrich Heine University Düsseldorf, Medical Faculty, Centre for Health and Society (chs), Institute of General Practice (ifam) , Düsseldorf, Germany

**Keywords:** general practice, teacher training, feedback, medical students, undergraduate medical education, evaluation

## Abstract

**Objectives:** The extent of university teaching in general practice is increasing and is in part realised with attachments in resident general practices. The selection and quality management of these teaching practices pose challenges for general practice institutes; appropriate instruments are required. The question of the present study is whether the student evaluation of an attachment in previously poorly evaluated practices improves after teaching physicians have received feedback from a colleague.

**Methods: **Students in study years 1, 2, 3 and 5 evaluated their experiences in general practice attachments with two 4-point items (professional competence and recommendation for other students). Particularly poorly evaluated teaching practices were identified. A practising physician with experience in teaching and research conducted a personal feedback of the evaluation results with these (peer feedback), mainly in the form of individual discussions in the practice (peer visit). After this intervention, further attachments took place in these practices. The influence of the intervention (pre/post) on student evaluations was calculated in generalised estimating equations (cluster variable practice).

**Results: **Of 264 teaching practices, 83 had a suboptimal rating. Of these, 27 practices with particularly negative ratings were selected for the intervention, of which 24 got the intervention so far. There were no post-evaluations for 5 of these practices, so that data from 19 practices (n=9 male teaching physicians, n=10 female teaching physicians) were included in the present evaluation. The evaluations of these practices were significantly more positive after the intervention (by n=78 students) than before (by n=82 students): odds ratio 1.20 (95% confidence interval 1.10-1.31; p<.001).

**Conclusion: **The results suggest that university institutes of general practice can improve student evaluation of their teaching practices via individual collegial feedback.

## Zusammenfassung

**Zielsetzung: **Die allgemeinmedizinische Lehre an den Universitäten nimmt zu und wird u.a. mit Praktika bei niedergelassenen Hausärzten realisiert. Auswahl und Qualitätsmanagement dieser Lehrpraxen stellen die allgemeinmedizinischen Institute vor Herausforderungen; entsprechende Instrumente sind gefragt. Die Fragestellung der vorliegenden Studie lautet, ob sich die studentische Bewertung eines Praktikums in bislang schlecht evaluierten Hausarztpraxen verbessert, nachdem die hausärztlichen Lehrärzte eine Rückmeldung durch eine Kollegin erhalten haben.

**Methodik: **Studierende der Studienjahre 1, 2, 3 und 5 bewerteten ihre Erfahrungen in hausärztlichen Praktika mit zwei 4-stufigen Items (fachliche Betreuung und Empfehlung für andere Kommilitonen). Besonders schlecht evaluierte Lehrpraxen wurden identifiziert. Eine praktisch tätige und lehr-erfahrene Hausärztin und wissenschaftliche Mitarbeiterin führte mit diesen eine persönliche Rückmeldung der Evaluationsergebnisse durch (Peer-Feedback), überwiegend in Form von Einzelgesprächen in der Praxis (peer visit). Nach dieser Intervention wurden in diesen Praxen weiter Praktika durchgeführt. Der Einfluss der Intervention (prä/post) auf die studentischen Evaluationen wurde in verallgemeinerten Schätzungsgleichungen (Clustervariable Praxis) berechnet.

**Ergebnisse: **Von insgesamt 264 Lehrpraxen hatten 83 eine suboptimale Bewertung. Davon wurden 27 besonders negativ bewertete Praxen für die Intervention ausgewählt, von denen in bislang 24 die Intervention umgesetzt werden konnte. Für 5 dieser Praxen gab es keine post-Evaluationen, so dass in die vorliegende Auswertung die Daten von 19 Praxen (n=9 männliche Lehrärzte, n=10 weibliche Lehrärztinnen) eingingen. Die Evaluationen dieser Praxen waren nach der Intervention (durch n=78 Studierende) signifikant positiver als vorher (durch n=82 Studierende): Odds Ratio 1.20 (95% Konfidenzintervall 1.10-1.31; p<.001).

**Schlussfolgerung: **Die Ergebnisse deuten darauf hin, dass allgemeinmedizinische Universitätsinstitute die studentische Bewertung ihrer Lehrpraxen über individuelle kollegiale Rückmeldungen verbessern können.

## Introduction

The German “Master Plan Medical Studies 2020” provides for a strengthening of the role of general practice in the curriculum [1]. One form of implementation desired by students and teachers is attachments in practices early and continuously in the course of studies [2]. Beyond pure learning effects, experiences that students make in these attachments can help shape a professional orientation. Good experiences in attachments can increase interest in general practice as a discipline and profession [3], [4]. 

In accordance with the Medical Licensing Regulations [https://www.gesetze-im-internet.de/_appro_2002/BJNR240500002.html], students in the Düsseldorf medical curriculum complete an attachment in general practices lasting a total of six weeks in the academic years 1, 2, 3 and 5 [https://www.medizinstudium.hhu.de]. The requirements of the attachments build on each other in terms of content; initially the focus is on anamnesis and physical examination, later more complex medical contexts and considerations for further diagnostics and therapy are added. Under the supervision of the resident teaching general practitioners (GP), the students can gain experience in doctor-patient interaction. An important and therefore repeatedly emphasised factor for a positive student perception of the attachments is the fact that the students are given the opportunity to work independently with patients during the attachment in order to be able to directly experience themselves in the provider role [2], [5]. The attitude and qualifications of the teaching physicians continue to play an important role in the didactic success of the attachments [3]. About 2/3 of the teaching practices are positively evaluated by the students, but about 1/3 are not. Due to the increasing demand for attachments in general practices since the installation of the new curriculum, many teaching practices have been newly recruited; a feedback culture is now being established. A first step was the possibility for teaching practices to actively request their written evaluation results, but this was almost never taken up. The next step of establishing a feedback strategy is reported here: One way to improve teaching performance is to receive feedback from an experienced colleague (peer feedback) [6]. This can generate insights that student evaluations alone cannot achieve and is increasingly recognised as a complement to student feedback. In personal peer feedback, ideas can be exchanged, problems discussed, strategies identified and concrete approaches to improvement found [7]. Potential effects include increased awareness and focus of the teaching physician on the teaching situation in practice, more information about what constitutes good teaching, motivation to be more interactive and student-centred, and inspiration to use new teaching methods [8]. Pedram et al. found positive effects on teacher behaviour after peer feedback, especially in terms of shaping the learning atmosphere and interest in student understanding [9]. The application of peer feedback to the setting described here has not yet been investigated. The research question of the present study is whether the student attachment evaluation of previously poorly rated GPs improves after peer feedback has been conducted.

## Methods

### Teaching practices

The data were collected during the 4 attachments in GP practices [https://www.uniklinik-duesseldorf.de/patienten-besucher/klinikeninstitutezentren/institut-fuer-allgemeinmedizin/lehre], all of which take place in teaching practices coordinated by the Institute of General Practice. Before starting their teaching practice, all teaching GPs are informed verbally and in writing about the collection of student evaluations and a personal interview with an institute staff member in case of poor evaluation results. 

Interested doctors take part in a 2-3 hour information session led by the institute director (SW) before taking up a teaching GP position, in which they are first informed about the prerequisites for teaching students in their practices. These include, among other things, the planning of time resources for supervising students in the attachments, enthusiasm for working as a GP, acceptance of the university’s teaching objectives in general practice (in particular that interns are allowed to work independently with patients) and participation in at least two of the eight didactic trainings offered annually by the institute (with the commencement of the teaching activity, the institute assumes the acceptance of these prerequisites on the part of the teaching physician, but does not formally check that they are met). This is followed by detailed information on the structure of the curriculum, the position of the attachments, the contents and requirements of the individual attachments and basic didactic aspects of 1:1 teaching. Information about the student evaluation of the attachment is provided verbally and in writing, combined with the offer to actively request both an overall evaluation and the individual evaluation by e-mail. There is no unsolicited feedback of the evaluation results to the practices. After the information event, a folder with corresponding written information is handed out.

Before each attachment, the teaching physicians are sent detailed material so that they can orient themselves once again. This contains information on the exact course of the attachment, on the current learning status of the students incl. enclosure of or reference to the underlying didactic materials, on the tasks to be worked on during the attachment and the associated learning objectives, on the relevance of practising on patients as well as a note on the attitude of wanting to convey a positive image of the GP profession to the students.

In addition, each student receives a cover letter to the teaching physician in which the most important points mentioned above are summarised once again.

#### Evaluation

Student evaluation as a regular element of teaching evaluation [https://www.medizin.hhu.de/studium-und-lehre/lehre] was carried out by independent student groups before and after the intervention. It consisted, among other things, of the opportunity for free-text comments, an indication of the number of patients personally examined and the items “How satisfied were you with the professional supervision by your teaching physician?” and “Would you recommend this teaching practice to other fellow students?” (both with a positively ascending 4-point scale). 

#### Selection of practices for the intervention

Since most practices received a very good evaluation (skewed distribution), three groups were identified as follows. From all the institute’s teaching practices involved in the attachments, those were first selected that had a lower than very good evaluation (=“suboptimal”): rated <2 at least once on at least one of the two above-mentioned items or repeatedly received negative free text comments. From this group of suboptimal (=less than very good) practices, those with more than two available student evaluations, continued teaching and particularly negative evaluations were selected: at least twice with <2 on at least one of the two items or repeated negative free text comments. Of the 27 practices, 24 practices (88.9%) have so far received an intervention to improve their teaching from a peer (n=3 not yet due to the pandemic), and 19 practices (70.4%) provided evaluation results from post-intervention attachments (n=5 had no attachments after the intervention). To characterise the three groups of very well, suboptimal and poorly evaluated (=selected) practices, an analysis of variance including post-hoc Scheffé tests was calculated with the factor group and the dependent variable evaluation result.

#### Intervention

Peer feedback was implemented as part of the didactic concept in particularly negatively evaluated teaching practices [https://www.uniklinik-duesseldorf.de/patienten-besucher/klinikeninstitutezentren/institut-fuer-allgemeinmedizin/didaktik-fortbildungen]: A GP staff member of the Institute of General Practice (EG) known to the teaching physicians and experienced in practice and teaching reported back to the teaching physicians their student evaluations. The primary mode was a personal visit to the practice (peer visit) [10]. For organisational reasons, group discussions with several teaching physicians and written feedback occasionally had to be offered as alternative solutions. Peer visits and group discussions were both aimed at reflecting on one's own teaching motivation and problems. This was followed by a discussion of the personal evaluation in order to enter into a constructive exchange between teaching GP and university with regard to teaching and dealing with students in the practice. Peer visits and group discussions were recorded. The opening question was “Why are you a teaching doctor?”, followed by questions about personal experiences: “Can you tell me about your experiences? What motivates you to be a teaching physician? Are there any problems from your point of view?” Then the (bad) feedback was addressed and discussed, followed by the question “What can we do to support you?”. The written feedback consisted of an uncommented feedback of the student evaluation results (scores and free texts). 

#### Analyses

Due to a strong correlation of the two evaluation items (Spearman's rho=0.79), these were averaged into an overall evaluation for the present analyses. In order to determine multivariable influences on this student evaluation, a generalised estimating equation (GEE) was calculated with the cluster variable “practice”, due to the lack of a normal distribution (Kolmogorov-Smirnov test p<.001) with gamma distribution and log linkage. The following were included as potential influence variables: Intervention effect (pre/post), intervention mode (peer visit vs. group/written), time of attachment (study year), number of patients seen in person per week. In parallel to this analysis, the intervention effect on the number of personally supervised patients was examined in a second GEE. 

The free texts in the student evaluations as well as the teacher comments in the peer visits and group discussions were processed qualitatively using content analysis in order to outline the underlying problems and the teacher reactions to the feedback in addition to the pure numbers. For this purpose, inductive category development was carried out on the material [11]. The numbers of negative student comments before and after the intervention were also compared quantitatively.

## Results

### Teaching practices and pre-evaluations

264 teaching practices with a total of 1648 attachments were involved. Of these, 181 practices (68.6%) with 1036 attachments were rated very good (student evaluation mean 3.8±standard deviation 0.2), 56 practices (21.2%) with 453 attachments were rated suboptimal (3.3±0.4) and 27 practices (10.2%) with 159 attachments were rated very poor (2.8±0.4). The overall comparison of the three groups shows significant differences (F(df=2)=205.1; p<.001), with significant differences in all post-hoc comparisons (all p<.001): very good vs. suboptimal (mean difference 0.51; standard error 0.04); very good vs. poor (1.09; 0.06); suboptimal vs. poor (0.58; 0.07). 

Table 1 (Tab. 1) describes the analysis sample of n=19 out of the 27 poorly rated practices in more detail.

Reasons for a poor evaluation according to free texts of the student evaluation can be presented in five categories. For example, the lack of opportunity to practise on patients was criticised.

*“Unfortunately, I did not have the opportunity to examine many patients myself during my last patient attachment, although I requested this on several occasions.” *(about practice ID 1) 

There were also comments about lack of appreciation and difficult communication:

*“The teaching doctor has little patience especially with foreign patients who cannot understand anatomical or medical terms. She makes insulting and ironic statements. With some patients I was left alone for 30 minutes while with others only 2 and afterwards she got annoyed when I was not done with the examination/anamnesis.”* (about practice ID 14)

Some teaching physicians were commented on with regard to their didactic competence: 

*“[…] as a teaching doctor, I experienced him as little to not at all competent and also very disinterested. He had no idea of what PA1 [Patient Attachment 1] was supposed to teach us and even after several approaches to him on my part, he understood little of what I was about or what I was supposed to learn there.”* (about practice ID 22)

Practice procedures and structures were mentioned which, according to the students, made it difficult to carry out the attachment efficiently:

*“From 8-11 am only patients come for blood collection, fixed appointments are not scheduled during that time. As I was not allowed to take blood or vaccinate, there was nothing for me to do during that time.”* (about practice ID 10)

In some practices with primarily non-German-speaking patients and also staff (incl. teaching physician), the language barrier turned out to be a problem in the evaluations. 

*“As the teaching doctor is [nationality XY], about 70% of the consultations were in [language XY].”* (about practice ID 2)

#### Intervention

In the protocols of the peer visits and group discussions with the teaching physicians, four categories of problems emerge, which partly mirror the student comments mentioned above: For example, the teaching physicians reported concerns about letting students work alone with patients (the following are quotes from the protocols of the intervening peer doctor.)

*“He finds it difficult to leave students alone. [...] He thinks the patients don’t like it that way, although his experience is actually different. Also has many patients from management. “Students are also too short in practice.”” *(reg. ID 17)

A sceptical attitude towards lower semester students in particular was also expressed.

*“Can’t do anything with the 2nd semesters, “they can’t do anything, there's no point in letting them listen to the heart if they don't know the clinical pictures”. [...] “The problem is also that they are always very young girls now.””* (reg. ID 24)

Some teaching physicians were not familiar with the didactic concepts and materials of the practical courses.

*“He has no knowledge of teaching, doesn't read through anything. Doesn’t know he is being evaluated either.”* (reg. ID 6)

In some cases, a self-image as a teaching general practitioner leads to the definition of one’s own attachment content, neglecting or devaluing the learning objectives set by the university. 

*““I’ve made a commitment to general practice and I want to pass that on”. Explains a lot to students, but doesn’t let them do much. “I show young people the right way. Nobody else does it (the university certainly doesn’t), so I do it.””* (reg. ID 4)

*“However, clearly wants to show the students everything, repeatedly mentions ultrasound, blood sampling, does not know teaching content, makes his own teaching content: “I show them everything of interest””.* (reg. ID 22)

At several points, the teaching physicians expressed intentions to change their behaviour, e.g. according to the minutes,* “wants to guide students more to examination”* or* “says he wants to read through the handouts in future”*. The majority of the teaching physicians showed a basic interest and commitment in supervising the students. Most were able to reflect on the points of criticism.

#### Pre-post analysis

The intervention effect on the student evaluation is significant and independent of the (also significant) influence of the number of patients (see table 2 (Tab. 2)).

The intervention effect on the number of patients personally cared for by students also persisted in a GEE (odds ratio 1.41; 95% confidence interval 1.21-1.64; p<.001), regardless of the type of intervention and study year (analysis not shown).

The proportion of critical comments in the student free-text comments decreases overall and in four of the five categories mentioned (see table 3 (Tab. 3)). 

## Discussion

In a pre-post comparison of poorly evaluated teaching physicians who supervised students in the context of GP attachments, peer feedback by a general practitioner had a positive effect on student evaluation and on the number of patients personally examined by students during the attachment. This is reflected in the evaluation scores and also in the fact that corresponding negative free-text comments by the students were less frequent after the intervention.

In line with the literature, it was crucial for student evaluation that students were given the opportunity to work independently with patients in order to experience themselves directly in the provider role [2], [5]. Also independent of the number of patients, student evaluation improved after the intervention: The qualitative results provide evidence that the teaching physicians may have been more closely engaged with the meaning of the attachments, the learning objectives and didactic materials after the intervention. This in turn also seemed to have had positive effects on the exchange and relationship between the teaching physician and the student (possibly in the sense of an alignment of mutual expectations) - also important elements of a positive attachment experience [3], [12]. The qualitative results on didactic competence and attitude indicate that, at least for the small group of previously poorly evaluated teaching physicians studied here, a more intensive consideration of their teaching assignment and repeated interaction between the university and the teaching practice is required in order to internalise contents and concepts and to implement them in the attachments for students in a recognisable and consistent manner. The fact that it is precisely the poorly evaluated teaching physicians who tend to rarely attend the meetings at the university (offered eight times a year in Düsseldorf) is an experience also reported by many other locations. The formal review of the prerequisites and criteria for an appropriate teaching GP position would involve an enormous amount of effort given the high number of teaching practices required – especially in a curriculum constructed along the lines of longitudinal general practice. However, it must be weighed up whether more resources should be invested in the selection and qualification of practices interested in teaching or in quality control and training of practices already teaching.

A strength of this study is the evaluations by independent student groups pre-post, so that biases due to repeated exposure of students to a practice (e.g. response shift bias, habituation, observer drift) are excluded. The weakness associated with the pre-post design without a control group and the focus on poorly evaluated practices is, among other things, the phenomenon of regression to the mean, which presumably accounts for part of the positive intervention effect. The primary research question of this study is formulated and answered quantitatively; we report only limited qualitative results. These allow only partial hypothesis-generating insights into the exact mechanisms of peer feedback [13]. In the present study, several modes of mediation of a peer feedback were realised. Since the analyses do not indicate different effects of the personnel and time-intensive peer visit on the one hand and the more efficient methods of group discussion and written feedback on the other, further studies are necessary to differentiate before a broader implementation. For example, Rüsseler et al. [14] found that written peer feedback – albeit in relation to lecturers – had positive effects on the design of the course.

## Conclusions

It makes sense to further consider the effects of teaching physician feedback in both research and teaching. The comprehensive GMA recommendations provide a robust framework for teaching [15] and the didactic qualification of teaching physicians [16]. Embedded in this, collegial peer feedback for poorly rated teaching physicians represents a possible tool for quality management of general practice teaching. 

## Competing interests

The authors declare that they have no competing interests. 

## Figures and Tables

**Table 1:**
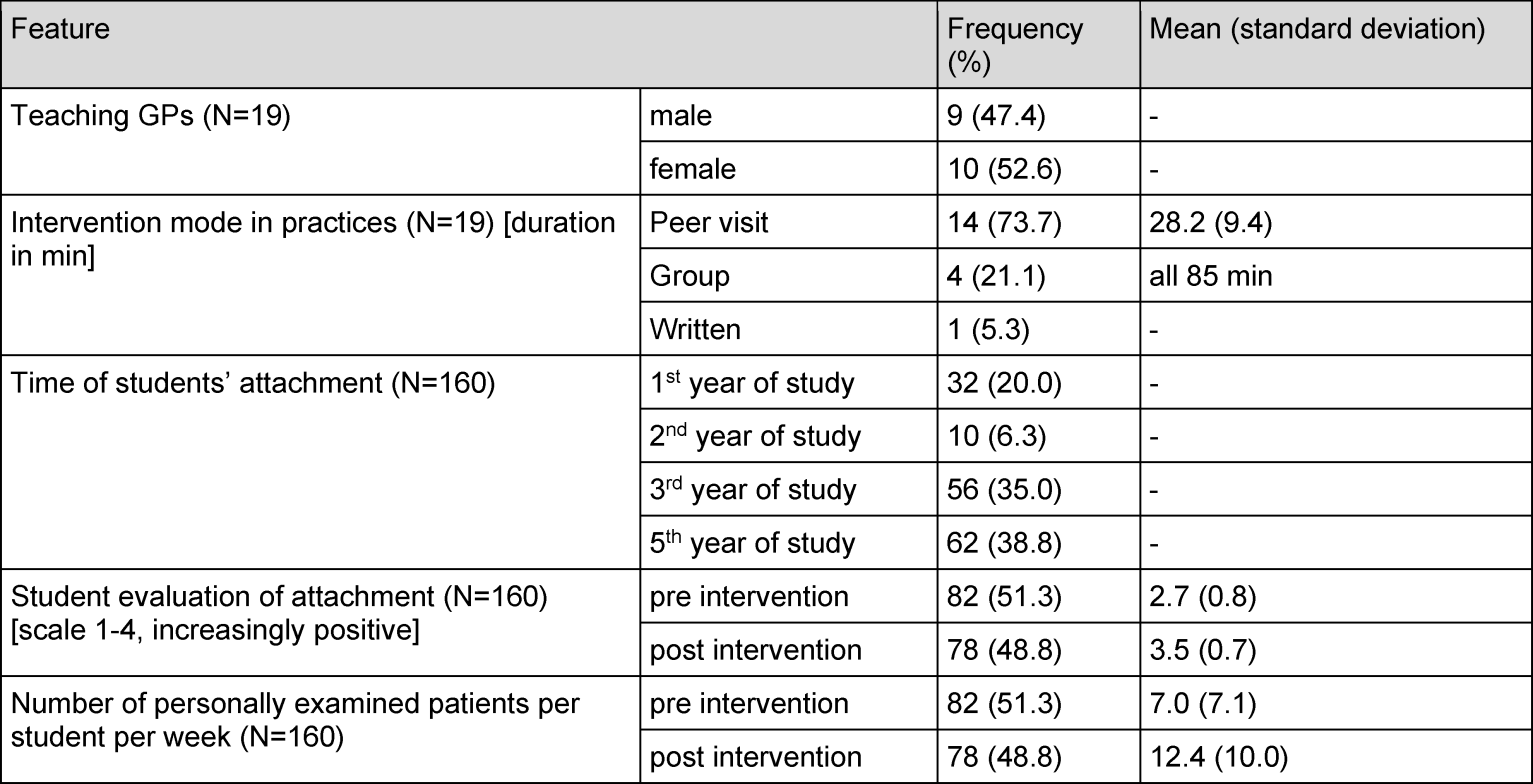
Characteristics of the analysis sample

**Table 2:**
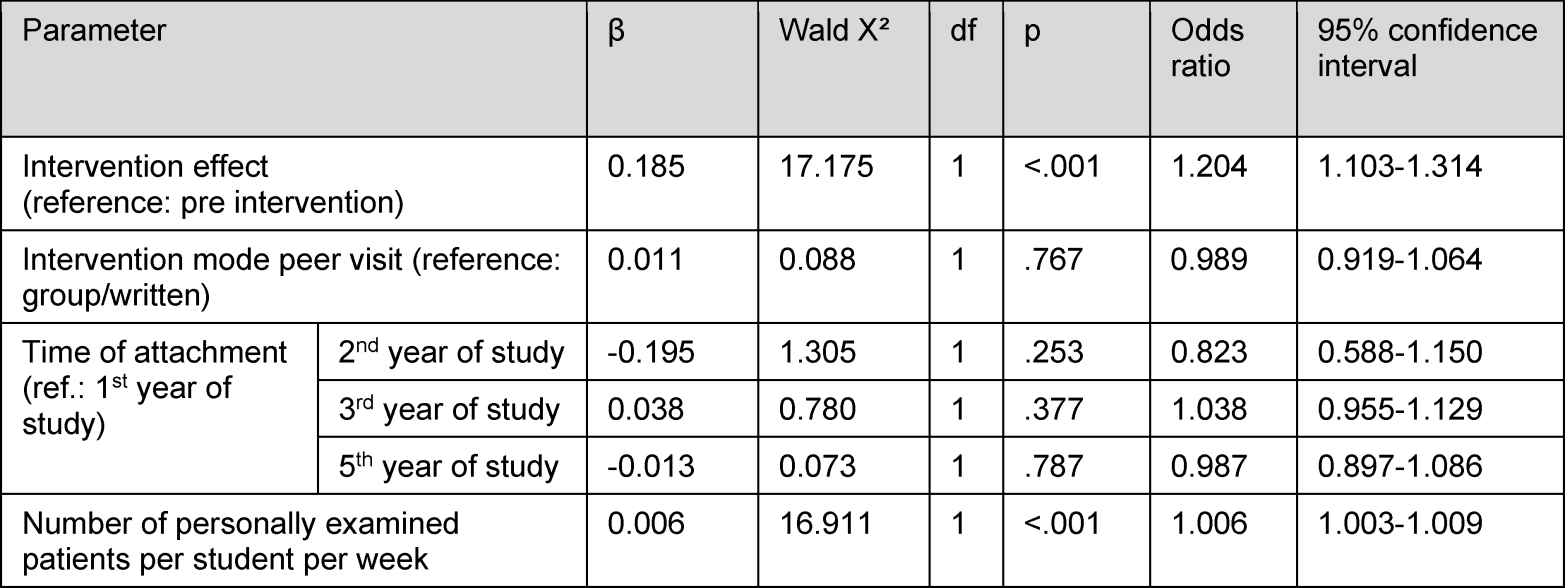
Multivariable influences on the dependent variable “student evaluation of GP attachment” (generalised estimating equation (GEE) with cluster variable practice)

**Table 3:**
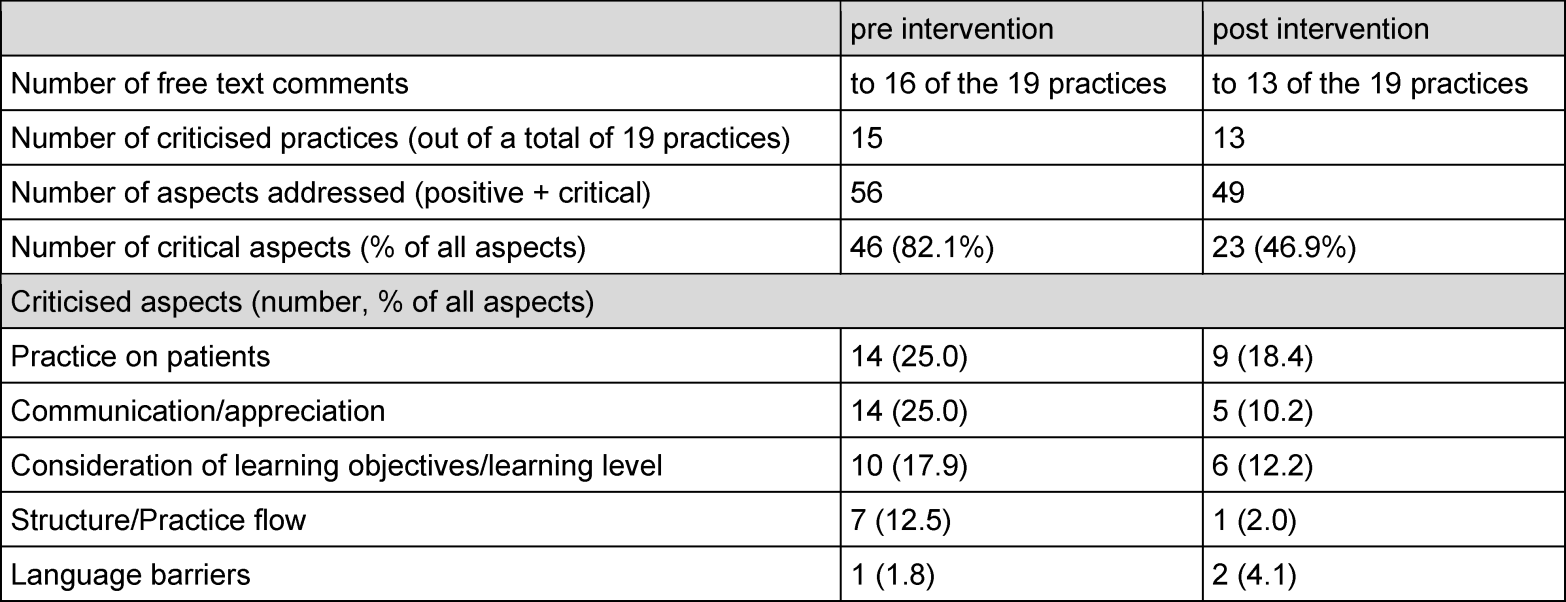
Number of students’ comments on attachments in 19 poorly evaluated GP teaching practices
